# Coronary and Peripheral Venous Inflammatory Biomarkers in Acute Myocardial Infarction: Associations with Myocardial Injury, Cardiac Function, and Clinical Outcomes

**DOI:** 10.3390/life16101594

**Published:** 2026-09-24

**Authors:** Mihai Melnic, Livia-Florentina Paduraru, Ioana-Antonia Lorent, Andreea-Catarina Popescu, Serban-Mihai Balanescu

**Affiliations:** 1 General Medicine Faculty, Department IV Cardio-Thoracic Pathology, Carol Davila University of Medicine and Pharmacy, 020021 Bucharest, Romania; 2 Cardiology Department, Elias Emergency University Hospital, 011461 Bucharest, Romania

**Keywords:** inflammation, myocardial infarction, biomarkers, interleukin 6

## Abstract

(1) Background: Interleukin-6 (IL-6) links innate immune activation to myocardial injury and adverse outcomes after acute myocardial infarction (AMI), yet the relationship between local intracoronary and systemic peripheral IL-6, and their prognostic value, remains unclear. (2) Methods: This prospective single-center study aimed to compare intracoronary and peripheral venous IL-6 and other inflammatory biomarkers in AMI, included 83 consecutive STEMI (ST-elevation myocardial infarction) or NSTEMI (non ST-elevation myocardial infarction) patients undergoing coronary angiography. Paired intracoronary and peripheral venous samples were obtained before revascularization and analyzed for IL-6, CRP, and ferritin, and correlated with LVEF (left ventricle ejection fraction), NT-proBNP, and a 30-day composite endpoint (death or heart failure hospitalization). (3) Results: Peripheral IL-6 exceeded intracoronary levels in 86.7% of patients (*p* < 0.001), with both peripheral IL-6 and the venous-to-coronary difference higher in STEMI than NSTEMI. IL-6 in both compartments correlated inversely with LVEF and positively with NT-proBNP and troponin I. Patients reaching the composite endpoint (10.8%) had markedly higher IL-6 and CRP. ROC analysis showed discriminatory ability for the endpoint by intracoronary and peripheral IL-6. (4) Conclusions: Intracoronary and peripheral IL-6 are associated with infarction phenotype, ventricular dysfunction, and short-term outcomes in AMI, supporting its role as an early prognostic biomarker.

## 1. Introduction

Acute myocardial infarction (AMI) remains one of the leading causes of morbidity and mortality worldwide despite substantial advances in reperfusion strategies and secondary prevention [1]. Beyond coronary artery occlusion, the extent of myocardial injury and the subsequent clinical outcome are largely influenced by the inflammatory response triggered after ischemia and reperfusion. In recent years, increasing evidence has highlighted inflammation not merely as a consequence of myocardial necrosis but as an active contributor to infarct expansion, adverse ventricular remodeling, and the development of heart failure [2,3,4,5]. Therefore, these observations have fueled the emergence of cardioimmunology as a rapidly evolving discipline investigating the bidirectional interactions between the immune and cardiovascular systems throughout the continuum of ischemic heart disease. Rather than viewing myocardial infarction solely as a consequence of coronary artery obstruction, the cardioimmunology paradigm recognizes AMI as a dynamic immunological process in which local vascular inflammation, innate and adaptive immune activation, and systemic immune responses collectively determine myocardial injury, infarct healing, ventricular remodeling, and long-term prognosis [3,6,7,8,9]. Among the numerous inflammatory mediators involved in the pathophysiology of AMI, interleukin-6 (IL-6) has emerged as a central cytokine linking innate immune activation with both local myocardial injury and systemic inflammatory responses. IL-6 is produced by a variety of cell types, including monocytes, macrophages, endothelial cells, fibroblasts, vascular smooth muscle cells, and cardiomyocytes in response to tissue injury [10,11]. In AMI, local ischemia triggers a cascade of cellular events, including disruption of cellular membrane integrity, increased oxidative stress, and cell death. These processes lead to the release of damage-associated molecular patterns (DAMPs), endogenous danger signals that alert the immune system and are recognized by pattern-recognition receptors (PRRs), including Toll-like receptors (TLRs) and NOD-like receptors (NLRs) expressed not only by immune cells but also by cardiomyocytes and endothelial cells [4,8,12,13]. Recognition of DAMPs by these receptors initiates and amplifies the inflammatory response, including activation of the NLRP3 inflammasome and subsequent activation of caspase-1, which cleaves the inactive precursors pro-IL-1β and pro-IL-18 into their biologically active forms [12,14,15,16]. IL-1β subsequently promotes the recruitment and activation of additional innate immune cells, thereby establishing a positive feedback loop that further amplifies the inflammatory response and promotes the production of additional pro-inflammatory cytokines, including IL-6, through activation of the NF-κB and mitogen-activated protein kinase (MAPK) pathways [13,14,17,18]. Thus, IL-6 expression rapidly increases within the infarcted myocardium and contributes to leukocyte recruitment, activation of the acute-phase response, endothelial dysfunction, and modulation of extracellular matrix remodeling [19,20,21,22]. Several clinical studies have demonstrated that elevated circulating IL-6 concentrations are associated with larger infarct size, reduced left ventricular systolic function, and an increased risk of adverse cardiovascular events following AMI [23,24]. Elevated IL-6 concentrations have also been investigated as a marker of residual cardiovascular risk in patients with established cardiovascular disease [25]. Moreover higher IL-6 levels have been correlated with impaired myocardial reperfusion, greater incidence of heart failure, recurrent ischemic events, and increased short- and long-term mortality [10,23,26,27] (Figure 1). Furthermore, IL-6 has attracted considerable interest as a potential therapeutic target, supported by evidence from anti-inflammatory trials suggesting that modulation of the IL-6 signaling pathway may improve cardiovascular outcomes in selected patient populations [28,29,30,31]. The inflammatory microenvironment surrounding the ruptured atherosclerotic plaque and the ischemic myocardium is characterized by intense local cytokine production, endothelial activation, platelet-leukocyte interactions, and microvascular dysfunction, processes that may not be fully captured by peripheral measurements [2,3,32]. Despite the recognized prognostic value of systemic inflammatory biomarkers, relatively little is known about the relationship between local intracoronary inflammatory activity and peripheral circulating cytokine levels during the acute phase of myocardial infarction. A limited number of studies have explored intracoronary cytokine profiles during AMI, and the relationship between local and systemic inflammatory responses remains incompletely understood [33,34]. Plaque-associated structural and extracellular-matrix alterations may also contribute to the local inflammatory milieu and mechanisms of plaque instability and erosion [35]. Coronary blood sampled directly from the culprit vessel may more accurately reflect the inflammatory milieu at the site of plaque rupture and myocardial ischemia, comparing intracoronary and peripheral inflammatory markers concentrations may provide additional insights into the spatial dynamics of inflammation during AMI and their potential clinical significance [20,33,36].

The present study was designed to investigate the inflammatory profile of patients presenting with acute myocardial infarction by simultaneously assessing IL-6 and other inflammatory biomarkers in peripheral venous blood and intracoronary arterial blood. By comparing cytokine concentrations between the two vascular compartments and evaluating their associations with left ventricular ejection fraction, NT-proBNP concentrations, and the occurrence of 30-day major adverse cardiovascular events, this study aims to clarify the prognostic significance of local and systemic inflammation during the early phase of myocardial infarction and to determine whether intracoronary IL-6 provides incremental information beyond conventional peripheral inflammatory assessment. A secondary aim was to assess the relationship between IL-6 and other inflammatory biomarkers, particularly CRP and ferritin, and to determine whether an intracoronary-to-peripheral difference of IL-6 is associated with clinical outcomes.

## 2. Materials and Methods

### 2.1. Study Design and Population

This single-center, prospective observational study was conducted at Elias University and Emergency Hospital, Bucharest, Romania, and included 83 consecutive patients presenting with acute myocardial infarction (AMI), including both ST-segment elevation myocardial infarction (STEMI) and non-ST-segment elevation myocardial infarction (NSTEMI), admitted between 8 July and 30 July 2026. The diagnosis of acute myocardial infarction was established according to the Fourth Universal Definition of Myocardial Infarction, based on clinical presentation, electrocardiographic findings, and elevated cardiac biomarkers [37]. All patients underwent coronary angiography, with percutaneous coronary intervention (PCI) performed when indicated according to the angiographic findings and clinical presentation.

Patients were excluded if they were younger than 18 years, unable or unwilling to provide informed consent, or had conditions that could substantially influence systemic inflammatory biomarker concentrations. These included active or recently diagnosed malignancy, active systemic inflammatory or autoimmune disease, acute or ongoing infectious disease, chronic inflammatory conditions with active disease, or other clinically significant conditions associated with substantial systemic inflammatory activation. Patients with clinical signs or symptoms suggestive of an acute infection at the time of admission were also excluded. As the study was conducted in a tertiary hospital providing care for adults, no upper age limit was applied.

Regarding the timing of coronary angiography, patients with STEMI underwent coronary revascularization within 12 h of symptom onset. Our center receives patients referred from a wide geographic area, with some patients transferred from hospitals located up to approximately 200–250 km away. Therefore, an exact symptom-to-angiography interval could not be reliably established for all patients, particularly because the precise time of symptom onset was not consistently documented. Coronary angiography was performed using a Philips Azurion 7 M20 angiographic system (Philips, the Nederland B.V. Veenpluis 6; manufactured in October 2022).

The study protocol complied with the Declaration of Helsinki and was approved by the Institutional Ethics Committee of the participating hospital. Written informed consent was obtained from all participants before enrollment (Ethics Committee of Elias University Emergency Hospital (No. 08072026-3/8 July 2026)).

### 2.2. Clinical Assessment

Baseline demographic characteristics, laboratory parameters, angiographic findings, and procedural characteristics were prospectively recorded. Clinical examination was performed at admission for all patients.

### 2.3. Blood Sampling and Laboratory Analysis

Paired blood samples were obtained from all 83 participants during coronary angiography. Peripheral venous blood was collected by standard venipuncture, while intracoronary arterial blood was obtained from the coronary ostium, proximal to the culprit lesion, before coronary intervention. In patients undergoing percutaneous coronary intervention, intracoronary blood was aspirated from the culprit coronary artery before balloon inflation or stent implantation, minimizing the potential influence of reperfusion on inflammatory marker concentrations. Paired samples were also obtained in the six patients who were subsequently referred for surgical rather than percutaneous revascularization. Inflammatory biomarkers concentrations—IL-6, C-reactive protein (CRP), ferritin—were measured in both coronary and peripheral venous blood using a standardized laboratory assay according to the manufacturer’s instructions. Intracoronary IL-6 refers to the measured IL-6 concentration in the intracoronary blood sample, expressed in pg/mL, whereas peripheral IL-6 refers to the corresponding concentration measured in peripheral venous blood. These measurements represent circulating IL-6 concentrations at the respective sampling sites and do not directly represent rates of IL-6 production or release). Interleukin-6 (IL-6) levels were measured by an electrochemiluminescence immunoassay (ECLIA) using the Elecsys IL-6 kit (Roche Diagnostics, Penzberg, Germany) on a Cobas e411 analyzer (Roche Diagnostics, Tokyo, Japan).

Additional laboratory parameters, including cardiac troponin I and N-terminal pro-B-type natriuretic peptide (NT-proBNP), were determined by the hospital’s central laboratory using routine standardized methods.

### 2.4. Echocardiographic Assessment

All patients underwent comprehensive transthoracic echocardiography within 24 h of admission, using standard imaging protocols. Left ventricular ejection fraction (LVEF) was calculated using the modified Simpson biplane method in accordance with current recommendations of the American Society of Echocardiography and the European Association of Cardiovascular Imaging, using a portable GE Vivid iq ultrasound system (GE HealthCare, 2024) [38].

### 2.5. Clinical Follow Up and Study Endpoints

Patients were followed for 30 days after the index myocardial infarction. Follow-up was performed through scheduled outpatient visits and/or structured telephone interviews conducted by the study investigators. Clinical outcomes occurring during the follow-up period were systematically recorded.

The primary clinical endpoint was a composite of hospitalization for heart failure and all-cause mortality occurring within 30 days after the index myocardial infarction. The associations between intracoronary and peripheral IL-6 concentrations, other inflammatory biomarkers, left ventricular systolic function assessed by LVEF, and NT-proBNP levels were evaluated in relation to the predefined clinical endpoint. A first clinical reassessment was performed at 30 days after the index hospitalization. The study is ongoing, with further clinical reassessments planned at 6 and 12 months.

### 2.6. Statistical Analysis

Continuous variables were assessed for normality using the Shapiro–Wilk test. Because inflammatory biomarkers showed markedly skewed distributions, continuous variables were summarized as median and interquartile range (IQR), and non-parametric tests were used for between-group comparisons. The Mann–Whitney U test was used to compare biomarker concentrations between STEMI and NSTEMI patients and between patients with and without 30-day endpoint. For each inflammatory biomarker, intracoronary and peripheral venous concentrations were analyzed separately. Because intracoronary and peripheral samples were obtained from the same patients, paired comparisons were performed using the Wilcoxon signed-rank test. The venous-to-coronary IL-6 gradient was calculated as the difference between peripheral venous and intracoronary concentrations (venous − intracoronary). Categorical variables were compared using Fisher’s exact test when expected cell counts were <5. Effect sizes for Mann–Whitney U comparisons were calculated as (r = |Z|/\sqrt{N}). Because multiple biomarker comparisons were performed, the Benjamini–Hochberg false discovery rate (FDR) procedure was applied to account for multiple testing. All statistical tests were two-sided, and a *p*-value < 0.05 was considered statistically significant. Given the limited number of 30-day composite clinical endpoint (mortality and re-hospitalization for heart failure), the prognostic analyses were considered exploratory, and no multivariable predictive model was constructed to avoid model overfitting. Statistical analyses were performed using IBM SPSS Statistics for Windows, Version 29.0 (IBM Corp., Armonk, NY, USA).

## 3. Results

### 3.1. Study Population and Baseline Features

A total of 83 patients with acute myocardial infarction were included in the study. The mean age was 57.55 ± 11.88 years (range, 30–80 years), and 66 patients (79.5%) were male. STEMI was the predominant clinical presentation, occurring in 65 patients (78.3%), whereas 18 patients (21.7%) presented with NSTEMI. The median BMI was 25.0 (22.0–31.0) kg/m^2^ in the STEMI group and 28.5 (22.0–31.0) kg/m^2^ in the NSTEMI group, with no statistically significant difference between the two groups (*p* = 0.644). Regarding baseline comorbidities and clinical characteristics assessed at the time of inclusion, hypertension was present in 56 (86.2%) patients in the STEMI group and 16 (88.9%) patients in the NSTEMI group, with no statistically significant difference between the groups (Fisher’s exact test, *p* = 1.000). Diabetes mellitus was diagnosed in 25 (38.5%) patients with STEMI and 8 (44.4%) patients with NSTEMI, with no significant difference between the groups (Pearson’s chi-square test, *p* = 0.646). Regarding BMI categories, 29 (44.6%) patients in the STEMI group and 6 (33.3%) patients in the NSTEMI group had normal weight, 14 (21.5%) and 3 (16.7%), respectively, were classified as overweight, while 22 (33.8%) and 9 (50.0%) were classified as obese. The distribution of BMI categories did not differ significantly between the two groups (Pearson’s chi-square test, *p* = 0.455). Regarding the extent of coronary artery disease, 30 patients (36.1%) had one-vessel disease, 28 (33.7%) had two-vessel disease, and 25 (30.1%) had three-vessel disease. Stent implantation was performed in 60 patients (72.3%), balloon angioplasty in 3 patients (3.6%), and combined balloon angioplasty and stent implantation in 14 patients (16.9%). Six patients (7.2%) underwent no revascularization procedure (Table 1).

**Table 1:** Baseline demographic, clinical, and angiographic characteristics of the study population (N = 83).

Parameter	Value
Age (years) mean +/− SD (range)	57.55 +/− 11.88 (30–80)
Male sex, n (%)	66 (79.5%)
Clinical presentation, n (%)	
STEMI	65 78.3%
NSTEMI	18 21.7%
Extend of coronary artery disease, n (%)	
One-vessel disease	30 (36.1%)
Two-vessel disease	28 (33.7%)
Three-vessel disease	25 (30.1%)
Revascularization procedure, n (%)	
Stent implantation	60 (72.3%)
Ballon angioplasty	3 (3.6%)
Ballon angioplasty + stent	14 (16.9%)
No revascularization	6 (7.2%)

STEMI, ST-elevation mocardial infarction; NSTEMI, non-ST-elevation myocardial infarction, Total N = 83 patients.

### 3.2. Intracoronary Versus Peripheral Venous IL6 Concentrations

Among the 83 patients, the median IL-6 concentration was 9.77 pg/mL (IQR, 5.98–19.27) in intracoronary samples and 15.34 pg/mL (IQR, 7.86–24.29) in peripheral venous samples. The distribution of IL-6 concentrations and paired differences was non-normal (Shapiro–Wilk test, *p* < 0.001); therefore, the Wilcoxon signed-rank test was used for paired comparisons. Peripheral venous IL-6 concentrations were higher than intracoronary concentrations in 72 patients (86.7%), whereas intracoronary concentrations were higher in 11 patients (13.3%); no ties were observed. The difference between peripheral venous and intracoronary IL-6 concentrations was statistically significant (Z = −6.561, *p* < 0.001) (Table 2).

**Table 2:** Comparison of coronary and peripheral venous IL-6 levels between patients with STEMI and NSTEMI.

IL-6 Parameter	STEMI, Median (IQR)	NSTEMI, Median (IQR)	U	*p*
Coronary IL-6 (pg/mL)	10.41 (13.38)	5.71 (11.70)	410.5	0.054
Peripheral venous IL-6 (pg/mL)	16.45 (19.82)	5.93 (13.47)	295.0	0.001
ΔIL-6 (venous-to-coronary)	4.07 (7.35)	0.41 (2.56)	202.0	<0.001

IQR, interquartile range; IL-6, interleukin-6; STEMI, ST-segment elevation myocardial infarction; NSTEMI, non-ST-segment elevation myocardial infarction; ΔIL-6, IL-6 gradient, analyzed as difference between venous to coronary value of IL-6.

### 3.3. Inflammatory Biomarkers According to Infarction Phenotype

Intracoronary IL-6 concentrations were higher in patients with STEMI than in those with NSTEMI, although the difference did not reach statistical significance (median [IQR], 10.41 [6.74–20.11] vs. 5.71 [4.24–15.94] pg/mL, respectively; Mann–Whitney U = 410.5, Z = −1.928, *p* = 0.054). In contrast, peripheral venous IL-6 concentrations were significantly higher in STEMI patients than in NSTEMI patients (16.45 [8.50–28.32] vs. 5.93 [4.06–17.53] pg/mL, respectively; U = 295.0, Z = −3.204, *p* = 0.001). The venous-to-coronary IL-6 gradient, calculated as peripheral venous minus intracoronary IL-6 concentration, was significantly greater in STEMI than in NSTEMI patients (median [IQR], 4.07 [7.35] vs. 0.41 [2.56] pg/mL, respectively; Mann–Whitney U = 202.0, Z = −4.232, *p* < 0.001; r = 0.465). In contrast, CRP concentrations did not differ significantly between STEMI and NSTEMI patients in either the intracoronary compartment (1.00 [11.05] vs. 3.10 [24.00] mg/L, respectively; *p* = 0.763) or the peripheral venous compartment (5.00 [14.50] vs. 5.25 [23.58] mg/L, respectively; *p* = 0.981). The venous-to-coronary CRP gradient also did not reach statistical significance (*p* = 0.054). Similarly, ferritin concentrations did not differ significantly between STEMI and NSTEMI patients in the intracoronary compartment (179.50 [194.00] vs. 144.75 [296.59] ng/mL, respectively; *p* = 0.765), peripheral venous compartment (187.00 [186.95] vs. 171.35 [315.27] ng/mL, respectively; *p* = 0.675), or for the venous-to-coronary gradient (*p* = 0.076). After adjustment for multiple comparisons using the Benjamini–Hochberg false discovery rate procedure, the differences in peripheral venous IL-6 concentrations and the venous-to-coronary IL-6 difference between STEMI and NSTEMI remained statistically significant (Table 3).

### 3.4. Associations Between IL-6 and Clinical and Cardiac Parameters

Intracoronary IL-6 concentrations showed weak positive correlations with age (Spearman’s ρ = 0.234, *p* = 0.033) and troponin I (ρ = 0.276, *p* = 0.012), as well as a positive correlation with NT-proBNP (ρ = 0.310, *p* = 0.004). Intracoronary IL-6 was inversely correlated with LVEF (ρ = −0.360, *p* = 0.001). Peripheral venous IL-6 concentrations were positively correlated with troponin I (ρ = 0.366, *p* = 0.001) and NT-proBNP (ρ = 0.346, *p* = 0.001), and inversely correlated with LVEF (ρ = −0.369, *p* = 0.001). The correlation between peripheral venous IL-6 and age did not reach statistical significance (ρ = 0.204, *p* = 0.065) (Figure 2, Table 3).

### 3.5. 30 Day Clinical Outcomes

During the 30-day follow-up period, 9 of 83 patients (10.8%) experienced the predefined 30-day composite clinical endpoint, consisting of death or hospitalization for heart failure. The endpoint occurred in 8 of 65 STEMI patients (12.3%) and in 1 of 18 NSTEMI patients (5.6%), with no statistically significant difference between the two infarction groups (Fisher’s exact test, *p* = 0.676). Patients who experienced the composite clinical endpoint had significantly higher IL-6 concentrations in both intracoronary and peripheral venous samples compared with those without an event (intracoronary: median [IQR], 125.90 [191.77] vs. 9.14 [9.75] pg/mL; U = 12.0, *p* < 0.001; r = 0.516; peripheral venous: 122.40 [309.14] vs. 13.03 [12.97] pg/mL; U = 22.0, *p* < 0.001; r = 0.500). In contrast, the venous-to-coronary IL-6 gradient value did not differ significantly between patients with and without an event (9.40 [124.65] vs. 2.74 [5.95] pg/mL; U = 246.0, *p* = 0.203). A similar pattern was observed for CRP. CRP concentrations were markedly elevated in patients who experienced the composite clinical endpoint, both in intracoronary samples (median [IQR], 75.60 [93.20] vs. 1.00 [9.63] mg/L; U = 65.0, *p* < 0.001; r = 0.470) and in peripheral venous samples (83.30 [147.80] vs. 1.00 [9.20] mg/L; U = 70.0, *p* < 0.001; r = 0.447), compared with patients without an event. The venous-to-coronary CRP diference did not differ significantly between the two groups (*p* = 0.501). Ferritin concentrations did not differ significantly between patients with and without the composite clinical endpoint in either the intracoronary compartment (257.10 [398.25] vs. 158.10 [200.18] ng/mL; *p* = 0.101) or peripheral venous compartment (293.90 [464.85] vs. 181.35 [195.83] ng/mL; *p* = 0.067). The venous-to-coronary ferritin gradient was also not significantly different between the two groups (*p* = 0.203). After Benjamini–Hochberg false discovery rate correction, the associations of intracoronary and peripheral venous IL-6 and CRP concentrations with the 30-day composite clinical endpoint remained statistically significant (Table 4).

#### ROC Analysis

An exploratory receiver operating characteristic (ROC) analysis showed that both intracoronary and peripheral venous IL-6 concentrations discriminated the 30-day composite clinical endpoint. Intracoronary IL-6 yielded an area under the curve (AUC) of 0.982 (95% CI, 0.955–1.000; *p* < 0.001); the exploratory threshold defined by Youden’s index (≥22.6 pg/mL) provided 100% sensitivity (95% CI, 66.4–100%) and 91.9% specificity (95% CI, 83.2–97.0%). Peripheral venous IL-6 had an AUC of 0.967 (95% CI, 0.921–1.000; *p* < 0.001); the corresponding threshold (≥81.7 pg/mL) provided 88.9% sensitivity (95% CI, 51.8–99.7%) and 97.3% specificity (95% CI, 90.6–99.7%). After bootstrap optimism correction, sensitivity was 95.1% for intracoronary and 83.4% for peripheral venous IL-6, and cross-validated sensitivity was 88.4% and 84.7%, respectively. When intracoronary IL-6 was dichotomized at ≥22.6 pg/mL, 9 of 15 patients (60.0%) with concentrations above the threshold experienced the 30-day composite clinical endpoint, compared with none of the 68 patients below it (Fisher’s exact test, *p* < 0.001; descriptive, as the threshold was derived from the same data). Given the small number of outcome events (n = 9) and the variability of the selected thresholds across resamples, the thresholds should be considered exploratory and hypothesis-generating, not clinical cut-off values, and require validation in an independent cohort (Figure 3).

## 4. Discussion

According to our results, we found that IL-6 concentrations differed significantly between peripheral venous and intracoronary samples, with higher peripheral venous concentrations in the majority of patients. Peripheral IL-6 and the venous-to-coronary IL-6 gradient were significantly higher in patients with STEMI than in those with NSTEMI. Both intracoronary and peripheral IL-6 concentrations were associated with lower LVEF and higher NT-proBNP levels. Most importantly, markedly higher intracoronary and peripheral IL-6 concentrations were observed in patients who experienced the 30-day composite clinical endpoint. The exploratory ROC analysis further supported the potential prognostic relevance of IL-6, with both intracoronary and peripheral concentrations showing discriminatory ability for the 30-day clinical endpoint. However, these findings should be interpreted cautiously given the small number of outcome events and the absence of an independent validation cohort. The identified thresholds should therefore be regarded as exploratory rather than definitive clinical cut-off values.

Several studies have demonstrated increased IL-6 production at the site of the culprit lesion following plaque destabilization and local myocardial ischemia, with higher IL-6 concentrations reported in coronary blood compared with peripheral blood, which contrasts with the findings of our study [34,39,40,41]. However, the timing of blood sampling appears to be an important determinant of this relationship. In particular, the largest differences between coronary and peripheral IL-6 concentrations have been reported approximately 6 h after symptom onset, whereas these differences appear to attenuate among patients presenting later after the onset of symptoms [39]. The timing between symptom onset and coronary angiography may represent a relevant factor when interpreting differences in IL-6 concentrations between peripheral venous and coronary blood. However, for our study, an exact symptom-to-angiography interval could not be reliably established for all patients in our cohort because of the wide geographic area from which patients were referred and the lack of precise documentation of symptom onset in some cases. Although differences in the concentration of the same biomarker between arterial and venous compartments may initially appear counterintuitive, acute myocardial infarction is characterized by marked endothelial and inflammatory instability, resulting in the episodic release of inflammatory mediators into the systemic circulation. This inflammatory response is dynamic and temporally heterogeneous rather than continuous and may therefore be characterized by intermittent systemic bursts of cytokine release [42,43]. During the early phase following myocardial infarction, IL-6 production may predominantly originate from the thrombosed culprit plaque because of local inflammation. Subsequently, ischemic and injured cardiomyocytes release damage-associated molecular patterns (DAMPs), which activate the innate immune system and promote the recruitment and activation of circulating inflammatory cells, including monocytes and macrophages. This systemic inflammatory response may subsequently contribute to increased circulating concentrations of inflammatory biomarkers [36,44]. The interpretation of the timing of symptom onset in our cohort requires caution. In particular, a substantial proportion of patients presented more than 6 h after symptom onset, which may have influenced the relative contribution of systemic and local inflammatory processes at the time of sampling. However, given the limitations in reliably establishing the exact time of symptom onset in all patients and the differences in the recommended timing of invasive management between STEMI and NSTEMI, symptom-to-sampling timing was not considered appropriate as a primary comparative variable in the present analysis. Another important consideration is the methodology used for coronary blood sampling. In our study, samples from the culprit vessel were obtained before revascularization, from blood located proximally to the coronary occlusion. Consequently, these samples may not fully reflect the local IL-6 concentration within the ischemic myocardium. The sampled blood likely represents a mixture of blood exposed to the culprit lesion and injured myocardial tissue with blood from the arterial circulation, potentially resulting in dilution of locally released IL-6. This methodological consideration may partly explain why higher IL-6 concentrations were not observed in culprit-lesion blood compared with peripheral blood in our cohort.

One of the key findings of our study was the association between IL-6 concentrations and both NT-proBNP levels and LVEF. Previous studies in patients with STEMI have demonstrated an independent association between IL-6 concentrations at presentation and infarct size, as well as left ventricular ejection fraction assessed by cardiac magnetic resonance imaging at 4 months, together with NT-proBNP concentrations [24,27]. Moreover, imaging-based assessments have demonstrated lower myocardial salvage following PCI and a greater extent of adverse ventricular remodeling in patients with higher inflammatory activity [27]. The association between increased IL-6 concentrations and impaired myocardial function may reflect both the extent of the initial myocardial injury and the subsequent contribution of inflammation to adverse ventricular remodeling. The association between increased IL-6 concentrations and impaired myocardial function may reflect both the extent of the initial myocardial injury and the subsequent contribution of inflammation to adverse ventricular remodeling [45,46]. Several mechanisms may account for the association between heightened inflammatory activity and post-infarction systolic dysfunction. These include endothelial dysfunction, ischemia–reperfusion injury, impaired contractile function of viable cardiomyocytes, and disruption of the normal myocardial healing process [45,46,47,48]. Persistent inflammatory activation may further promote excessive fibrosis and adverse left ventricular remodeling, ultimately leading to ventricular dilatation and progressive deterioration of cardiac function [2,23,24,49,50,51,52].

The positive association between IL-6 concentrations and troponin I supports an association between inflammatory activity and the extent of acute myocardial injury. In our cohort, both intracoronary and peripheral venous IL-6 concentrations were positively correlated with troponin I, although the association was somewhat stronger for peripheral venous IL-6 (ρ = 0.366) than for intracoronary IL-6 (ρ = 0.276). As troponin I reflects cardiomyocyte injury and necrosis, the observed association with IL-6 suggests that higher inflammatory activity may be present in patients with greater myocardial injury during the acute phase of myocardial infarction. However, these associations should not be interpreted as evidence that IL-6 directly mediates myocardial injury, as the observational design of the present study does not allow causal inferences [23,24]. Importantly, the presence of this association in both intracoronary and peripheral samples indicates that the observed relationship between IL-6 concentrations and myocardial injury is detectable not only in the local coronary circulation but also in the systemic circulation. Nevertheless, given the observational nature of these associations, the directionality and underlying mechanisms cannot be determined from the present study. Higher IL-6 concentrations may reflect the inflammatory response accompanying more extensive myocardial injury, while the possibility of reciprocal interactions between myocardial injury and inflammatory activation cannot be excluded. These interpretations should therefore be considered hypothesis-generating rather than evidence of a direct causal role of IL-6 in myocardial injury.

Closely related to the association between IL-6 and LVEF was the positive association observed between IL-6 concentrations and major adverse clinical events at 30 days (all-cause mortality and hospitalization for heart failure). This finding is consistent with observations from the SOLID-TIMI 52 study, which also reported an association between inflammatory biomarkers and clinical outcomes in patients with acute myocardial infarction [10]. Several mechanisms have been proposed in the literature that may help explain the association between elevated IL-6 concentrations and adverse clinical outcomes. Persistent systemic inflammatory activity may be associated with greater atherosclerotic plaque instability and an increased risk of recurrent ischemic events. Similarly, inflammatory activation has been implicated in extracellular matrix degradation and adverse tissue remodeling through increased matrix metalloproteinase activity, as well as in alterations of myocardial healing. These processes have been proposed as potential contributors to myocardial fibrosis, increased ventricular stiffness, elevated filling pressures, and the development or progression of heart failure [6,24,27,53,54]. The prognostic relevance of IL-6 is further supported by meta-analyses demonstrating an association between elevated IL-6 concentrations and long-term MACE (major adverse cardiac events), with a substantial proportion of adverse events occurring during the first year following AMI [27,45]. The association between elevated IL-6 levels and mortality may also reflect, at least in part, the greater burden of comorbidities observed among patients with higher IL-6 concentrations, including hypertension, peripheral arterial disease, and chronic kidney disease. However, these potential mechanisms cannot be established from the present observational study, and the observed associations should not be interpreted as evidence of a direct causal effect of IL-6 on clinical outcomes [10,26,55].

An exploratory ROC analysis suggested that both intracoronary and peripheral venous IL-6 concentrations discriminated the 30-day composite endpoint. However, the thresholds were selected and evaluated in the same small dataset (9 events), and internal validation indicated that performance estimates were optimistic: the sensitivity of the intracoronary threshold decreased from 100% to 95.1% after bootstrap optimism correction and to 88.4% in cross-validation, and the selected thresholds varied considerably across resamples, particularly for peripheral venous IL-6. These thresholds should therefore be regarded as exploratory and hypothesis-generating, not as clinical cut-off values, and require validation in an independent cohort.

Taking all these findings into consideration, IL-6 concentration may provide information related to the extent of myocardial injury and systemic inflammatory activity during AMI, while their interpretation appears to depend on the timing and site of blood sampling. However given the observational design of the present study, the observed associations should not be interpreted as evidence of a direct causal or prognostic effect of IL-6. Recent data from the ZEUS trial further highlight the complexity of targeting inflammation to reduce cardiovascular events. This randomized, double-blind trial investigated ziltivekimab, a monoclonal antibody targeting the IL-6 pathway, in patients with established atherosclerotic cardiovascular disease and chronic kidney disease [54]. Despite effectively reducing circulating IL-6 and high-sensitivity C-reactive protein concentrations, ziltivekimab did not reduce the incidence of MACE compared with placebo (HR 0.99, 95% CI 0.88–1.11) [55,56]. These findings illustrate that modulation of a circulating inflammatory pathway does not necessarily translate into a reduction in clinical events and underscore the complexity of the relationship between inflammatory biomarkers and cardiovascular outcomes [57,58]. Systemic inflammatory biomarkers may reflect multiple biological processes occurring at both local and systemic levels, and circulating IL-6 concentrations may not fully characterize inflammatory activity within the culprit coronary lesion or myocardial tissue. [59,60,61]. In this context, the distinct relationships observed between intracoronary and peripheral IL-6 concentrations in our study highlight the potential importance of sampling site when investigating inflammatory responses during AMI. Further studies examining differences between intracoronary and peripheral IL-6 concentrations and their temporal changes across different phases of AMI may help clarify the relationship between local and systemic inflammatory activity and determine whether these measurements provide additional biological or prognostic information beyond circulating IL-6 concentrations.

## 5. Limitations

Before drawing conclusions from the present study, several limitations should be acknowledged. First, the relatively small sample size limits the statistical power to detect significant differences between the analyzed subgroups. Consequently, potentially relevant associations between patient’s clinical and biological features and their prognosis may have remained undetected, while other associations may not have reached the statistical significance required to support definitive conclusions. Second, the relatively low number of adverse events observed during follow-up limits the assessment of the role of IL-6 in their occurrence. The possibility that unmeasured or insufficiently accounted-for confounding factors contributed to the observed outcomes cannot be excluded, thereby reducing the robustness of the prognostic associations identified. Third, the relatively short follow-up period may have limited the detection of later adverse cardiovascular events and, consequently, the ability to fully assess the long-term prognostic significance of inflammatory biomarkers. Longer follow-up periods would be valuable to determine whether the associations observed in the early post-infarction setting persist over time and whether IL-6 may have a sustained prognostic role. Fourth, the single-center design may limit the generalizability of the findings, as the results may have been influenced by the demographic and clinical characteristics of the study population, center-specific protocols, the methods used for the assessment of clinical, biological and echocardiographic parameters, and the experience of the medical staff. Fifth, the study design did not include sampling from the coronary venous circulation, which could have provided additional insight into the local inflammatory response associated with myocardial infarction by capturing a mixture of cytokines released from both the culprit lesion and the ischemic myocardium [62]. Sixth, the lack of serial measurements of inflammatory biomarkers limits the assessment of the temporal dynamics of the inflammatory response throughout the course of AMI. Therefore, the present study should be regarded as hypothesis-generating, with its findings warranting confirmation in larger, multicenter studies with longer follow-up periods and serial sampling of multiple inflammatory biomarkers. Future investigations should also explore transcoronary gradients between coronary venous and arterial blood, as well as between coronary venous and peripheral venous blood. A more comprehensive approach may provide a better characterization of the inflammatory response following AMI, help identify the optimal timing and sampling site for biomarker assessment, and further clarify the potential role of IL-6 as an independent predictor of adverse cardiovascular events.

## 6. Conclusions

In patients with acute myocardial infarction, IL-6 concentrations were strongly associated with infarction phenotype, ventricular dysfunction, and adverse clinical outcomes at 30 days. Both intracoronary and peripheral IL-6 showed potential prognostic relevance, whereas the venous-to-coronary gradient itself was not associated with the clinical endpoint. These findings do not support the current use of IL-6 for routine diagnostic or risk stratification purposes, but rather provide hypothesis-generating evidence regarding the potential biological and prognostic relevance of IL-6 and the importance of vascular sampling site in AMI. These findings support further investigation of IL-6 as an early inflammatory biomarker in AMI, particularly in larger prospective multicenter cohorts with longer follow-up and independent validation of clinically relevant thresholds.

## Figures and Tables

**Figure 1:**
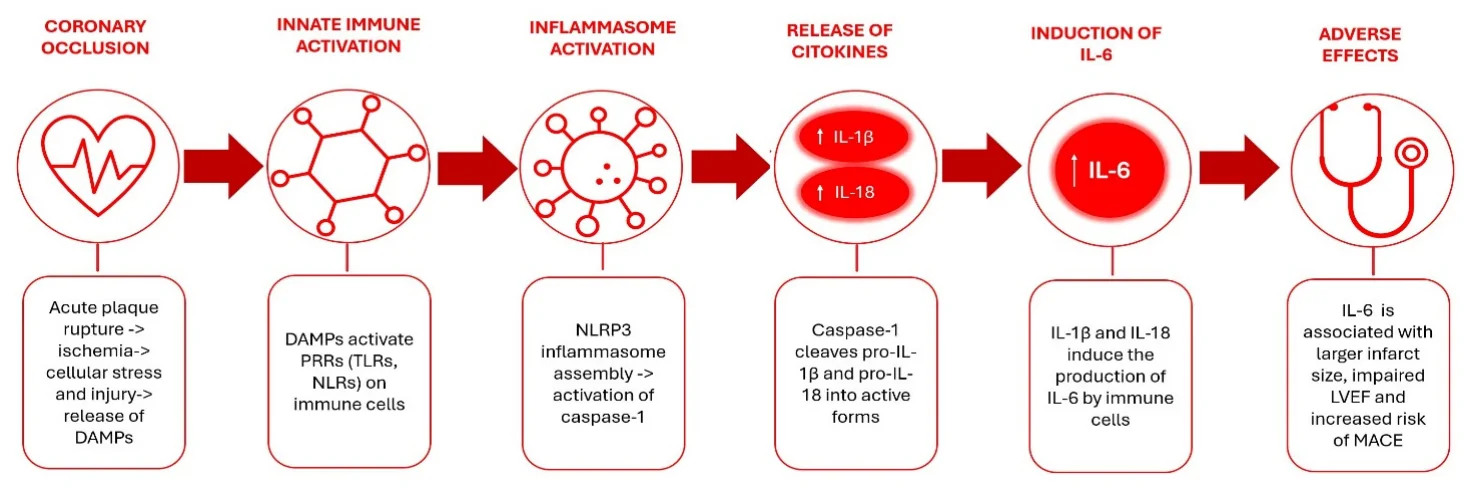
**Pathophysiological Pathways of IL-6 Activation in Acute Myocardial Infarction.** *DAMPs, damage-associated molecular patterns, PRRs, pattern-recognition receptors TLRs, Toll-like receptors, NLRs, NOD-like receptors, NLRP3, NOD-, LRR- and pyrin domain-containing protein 3, IL, interleukin, LVEF, left ventricular ejection fraction, MACE, major adverse cardiovascular events.*

**Figure 2:**
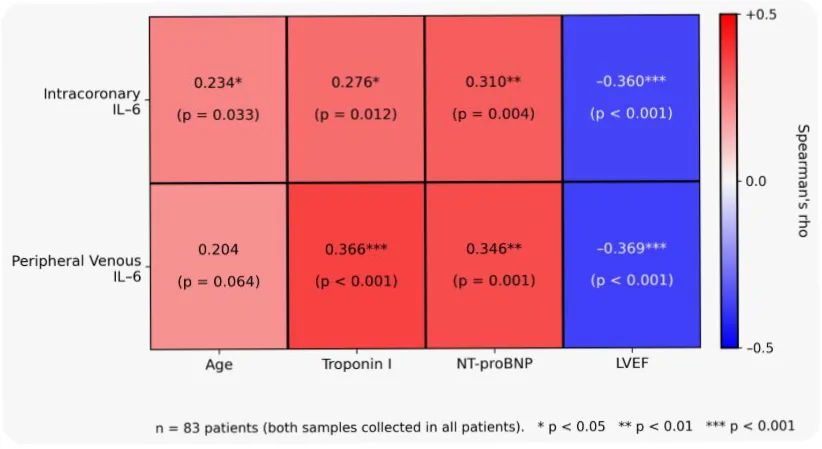
***Associations between IL-6 and Clinical and Cardiac Parameters.*** Heatmap of Spearman’s rank correlation coefficients (rho) between interleukin-6 (IL-6) levels measured in intracoronary and peripheral venous blood and age, troponin I, N-terminal pro-B-type natriuretic peptide (NT-proBNP), and left ventricular ejection fraction (LVEF) (n = 83 patients). Each cell shows rho with the exact p-value in parentheses. Colour indicates the direction and strength of the correlation (red, positive; blue, negative). * p < 0.05, ** p < 0.01, *** p < 0.001.

**Figure 3:**
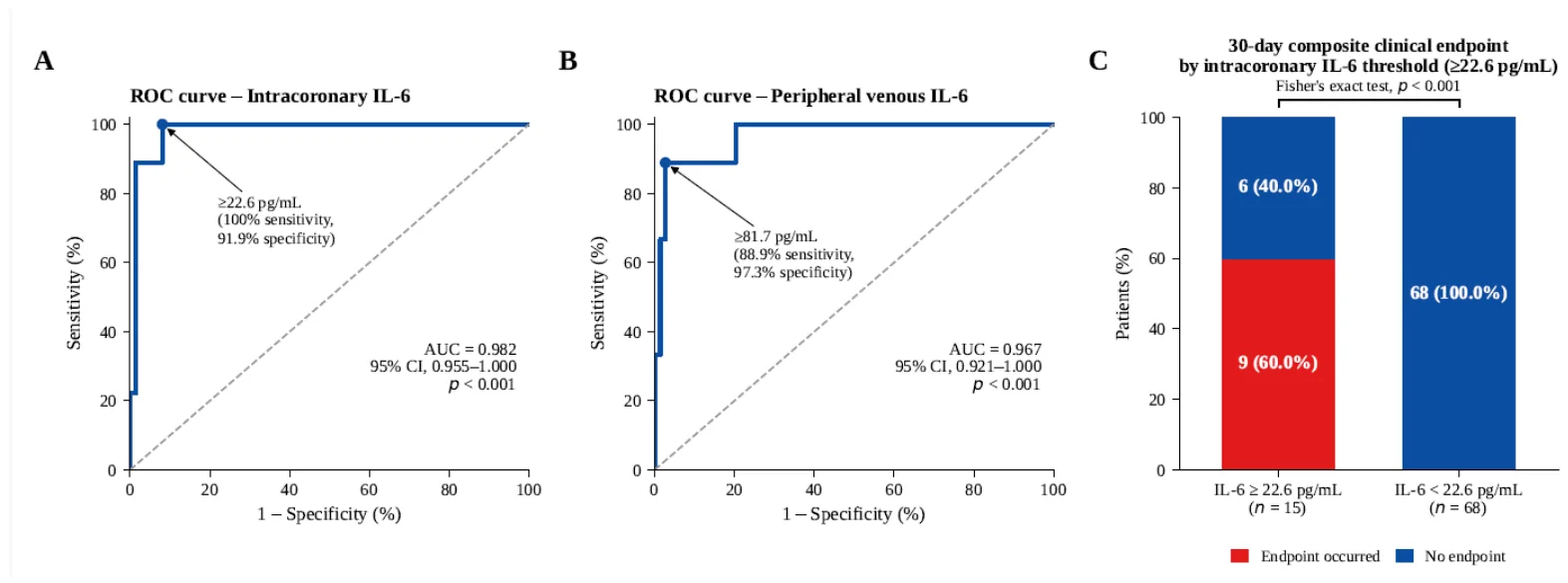
***Discrimination** **of Intracoronary and Peripheral Venous IL-6 for the 30-day Composite Clinical Endpoint.*** *Discrimination of intracoronary and peripheral venous IL-6 for the 30-day composite clinical endpoint. (**A**) ROC curve for intracoronary IL-6 (AUC 0.982; 95% CI, 0.955–1.000; p < 0.001); the threshold maximizing Youden’s index (≥22.6 pg/mL) provided 100% sensitivity and 91.9% specificity. (**B**) ROC curve for peripheral venous IL-6 (AUC 0.967; 95% CI, 0.921–1.000; p < 0.001); the threshold maximizing Youden’s index (≥81.7 pg/mL) provided 88.9% sensitivity and 97.3% specificity. (**C**) Incidence of the endpoint according to the intracoronary threshold (Fisher’s exact test, descriptive). Given the small number of events (n = 9), thresholds were selected and evaluated in the same dataset and should be considered exploratory.*

**Table 3:** Comparison of LVEF and selected biomarkers between STEMI and NSTEMI groups.

Variable	STEMI (n = 65)	NSTEMI (n = 18)	*p*-Value
LVEF, %	40.0 (35.0–48.5)	50.0 (45.0–51.25)	<0.001
Troponin I (ng/mL)	43.8 (16.45–80.00)	3.11 (0.44–9.72)	<0.001
NT-proBNP (pg/mL)	2430 (675–8950)	732.7 (378.2–4900)	0.036
CRP mg/L	5.0 (1.0–15.5)	5.25 (1.0–24.58)	0.981

*Data are presented as median (IQR). Comparisons between STEMI and NSTEMI groups were performed using the Mann–Whitney U test. STEMI, ST-segment elevation myocardial infarction; NSTEMI, non-ST-segment elevation myocardial infarction; LVEF, left ventricular ejection fraction; NT-proBNP, N-terminal pro-B-type natriuretic peptide; CRP, C-reactive protein.*

**Table 4:** Comparison of inflammatory biomarkers levels in coronary and peripheral blood and their association with 30-day adverse clinical events.

Biomarker	Sampling Site/Comparison	Mean Rank No 30-Day Adverse Clinical Events, n (%)	Mean Rank 30-Day Adverse Clinical Events, n (%)	U	Z	*p*	r
IL-6 (pg/mL)	Coronary	37.66	**77.67**	12.0	−4.701	**<0.001**	**0.516**
	Peripheral venous	37.80	**76.56**	22.0	−4.555	**<0.001**	**0.500**
	Venous − coronary	40.82	51.67	246.0	−1.274	0.203	0.140
CRP (mg/mL)	Coronary	38.38	**71.78**	65.0	−4.281	**<0.001**	**0.470**
	Peripheral venous	38.45	**71.22**	70.0	−4.069	**<0.001**	**0.447**
	Venous − coronary	41.41	46.83	289.5	−0.673	0.501	0.074
Ferritin (ng/mL)	Coronary	40.49	54.44	221.0	−1.640	0.101	0.180
	Peripheral venous	40.31	55.89	208.0	−1.831	0.067	0.201
	Venous − coronary	40.82	51.67	246.0	−1.274	0.203	0.140

*CRP, C-reactive protein; IL-6, interleukin-6; IQR, interquartile range; 30-day adverse clinical events were defined as all-cause mortality or hospitalization for heart failure. Note: Values are presented as median (IQR). The venous-to-coronary gradient was calculated as peripheral venous concentration minus coronary concentration. p-values were obtained using the two-sided Mann–Whitney U test. Effect size was calculated as r = ∣ Z ∣ /N. Bold p-values remained statistically significant after Benjamini–Hochberg FDR correction.*

## Data Availability

The data presented in this study are available on reasonable request from the corresponding author. The data are not publicly available due to privacy and ethical restrictions.
